# Supplementary material for: MicroRNA-200c-5p Regulates Migration and Differentiation of Myoblasts via Targeting Adamts5 in Skeletal Muscle Regeneration and Myogenesis
Source: Int J Mol Sci. 2023 Mar 5;24(5):4995. doi: 10.3390/ijms24054995 (PMC10003123; doi:10.3390/ijms24054995)
Supplement: Supplementary file 1 [file ijms-24-04995-s001.zip › Supplementary Figure.pdf]

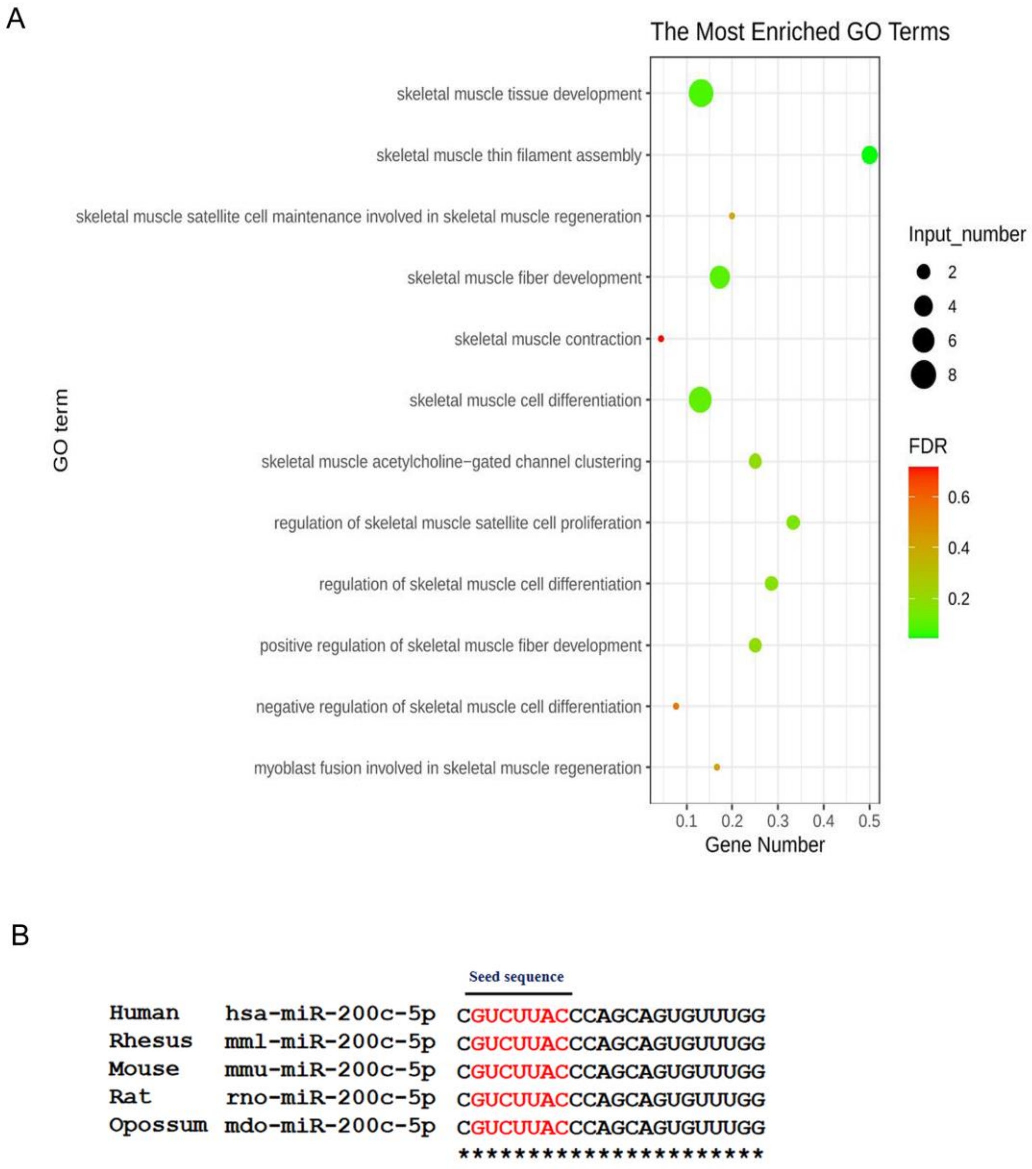

Figure S1. Potential target genes and interspecies conserved of miR-200c-5p. **A.** GO analysis of Targetscan results showed that the predicted target genes of miR-200c-5p were involved in skeletal muscle regeneration and development. **B.** Conservation of miR-200c-5p among different species.

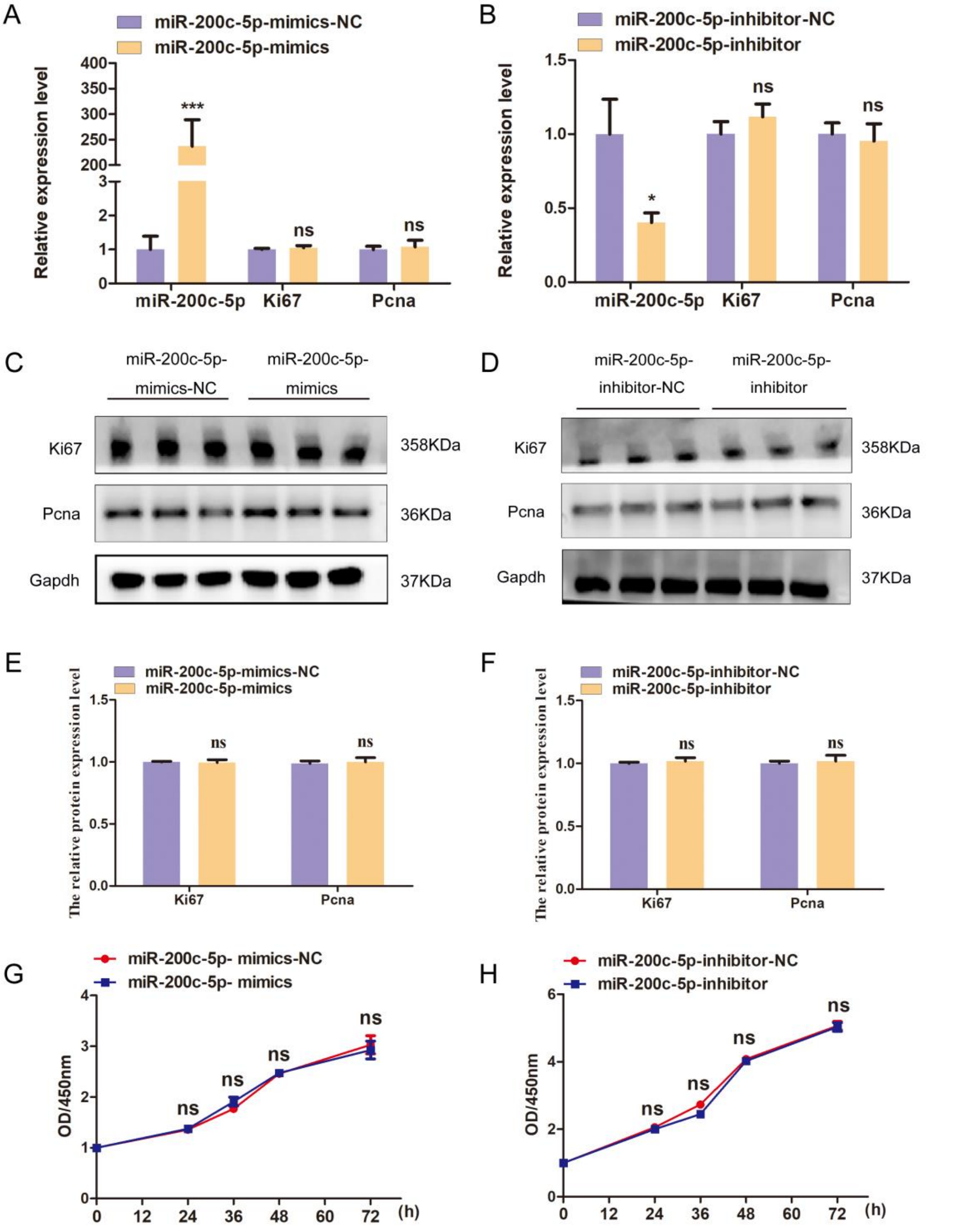

Figure S2. miR-200c-5p does not affect the proliferation of C2C12 myoblast. Effects of miR-200c-5p mimics (**A**) and inhibitor (**B**) on mRNA levels of Pcna and Ki67. Effects of miR-200c-5p mimics (**C**) and inhibitor (**D**) on protein levels of Pcna and Ki67. **E**.and **F.** The protein gray value was evaluated by Image J. CCK-8 assay showed that there was no difference in the number of miR-200c-5p mimics (**G**) and inhibitor (**H**) groups at 12h, 24h, 36h, 48h, and 72h. All experiments were repeated at least three times, Gapdh was used for normalization. The data are presented as Mean ± S.E.M. * $p < 0.05$, ** $p < 0.01$, *** $p < 0.001$.

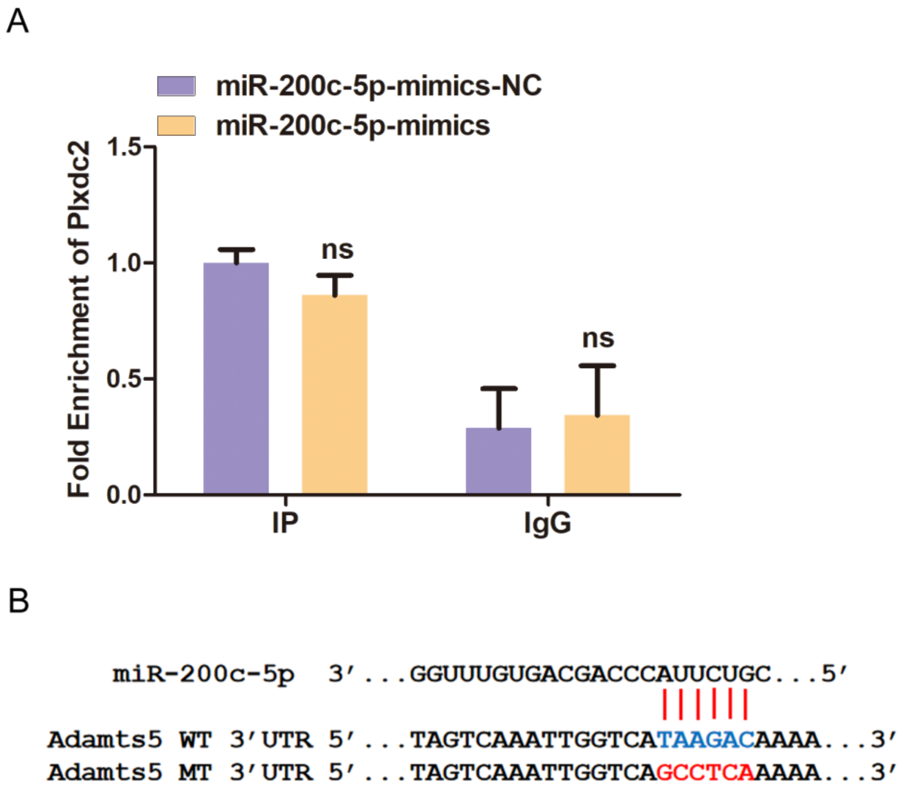

Figure S3. *Plxdc2* in RIP experiment and 3'UTR of *Adamts5*. **A.** The enrichment multiples of Plxdc2 in RIP experiment. **B.** Schematic diagram of the binding site and mutation site of miR-200c-5p on *Adamts5* 3'UTR. .

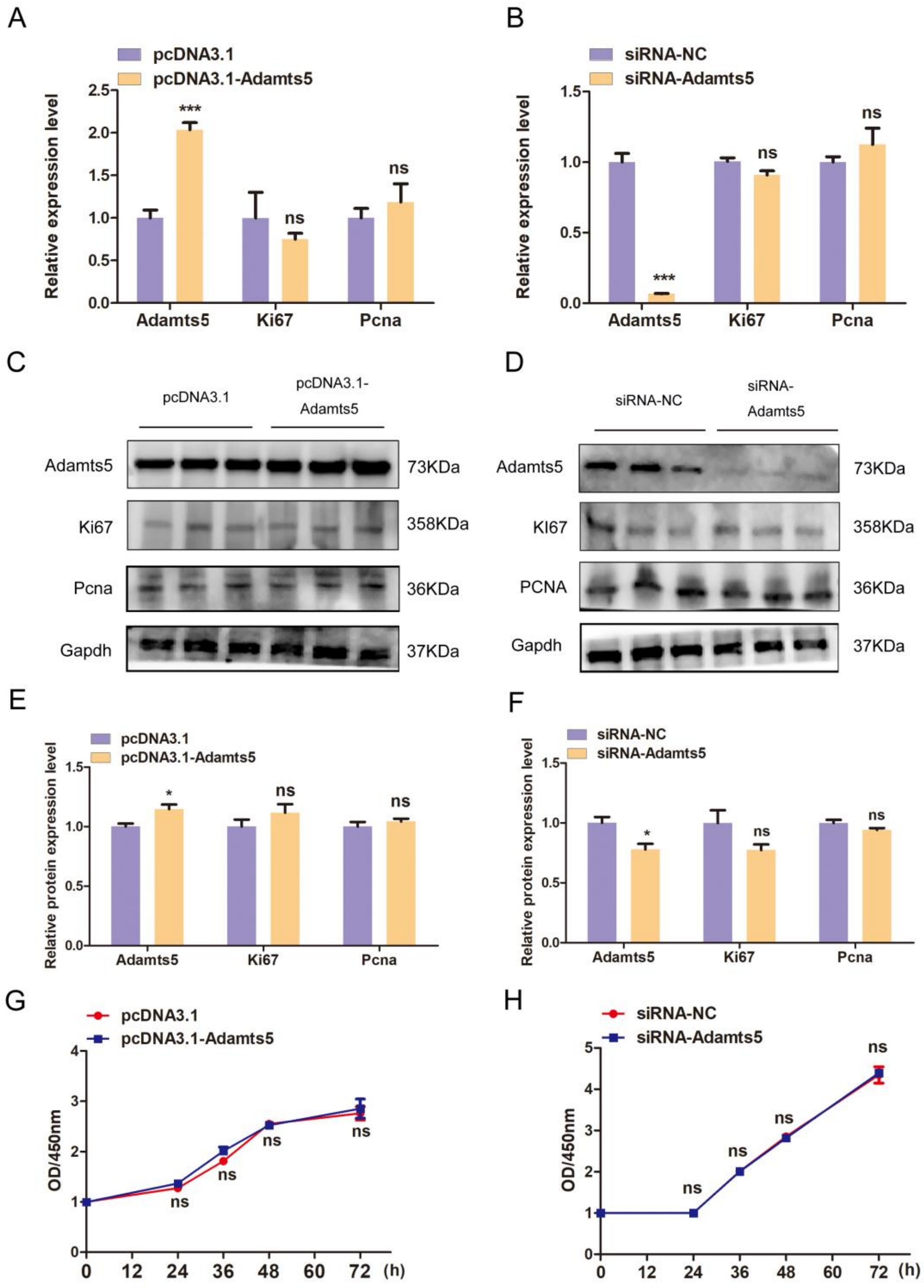

Figure S4. *Adamts5* does not affect the proliferation of C2C12 myoblast. Effects of *Adamts5* overexpression (**A**) and knockdown (**B**) on mRNA levels of Pcna and Ki67. Effects of Adamts5 overexpression (**C**) and knockdown (**D**) on protein levels of Pcna and Ki67. **E.**and **F**. The protein gray value was evaluated by Image J. CCK-8 assay showed that there was no difference in the number of *Adamts5* overexpression (**G**) and knockdown (**H**) groups at 12h, 24h, 36h, 48h, and 72h. All experiments were repeated at least three times, Gapdh was used for normalization. The data are presented as Mean ± S.E.M. * $p < 0.05$, ** $p < 0.01$, *** $p < 0.001$.
