# Supplementary material for: MicroRNA-200c-5p Regulates Migration and Differentiation of Myoblasts via Targeting Adamts5 in Skeletal Muscle Regeneration and Myogenesis
Source: Int J Mol Sci. 2023 Mar 5;24(5):4995. doi: 10.3390/ijms24054995 (PMC10003123; doi:10.3390/ijms24054995)
Supplement: Supplementary file 1 [file ijms-24-04995-s001.zip › Supplementary Table.pdf]

Table S1. Oligonucleotides used in this study

Name	***Sequence***
miR-200c-5p-mimics	CGUCUUACCCAGCAGUGUUUGG
miR-200c-5p-inhibitor	CCAAACACUGCUGGGUAAGACG
si-m-Adamts5_001	GTACATGATTTCCACTTCA
si-m-Adamts5_002	CCCAAGAAGACCACTCAAA

Table S2. qPCR primers used in this study

Gene	***Primer Sequence (5'-3')***
MyHC	F:AGCAGCGACACTGAAATGGA
	R:GTTGTCGTTCCTCACGGTCT
MyoG	F:GCCCAGTGAATGCAACTCCCACA
	R:CAGCCGCGAGCAAATGATCTCCT
MyoD	F:CGAGCACTACAGTGGCGACTCAGAT
	R:GCTCCACTATGCTGGACAGGCAGT
Plac8	F:CAGTGGAGCACAGCAGACTT
	R:TCCGCAGTCACTGAAGCAAT
Eno1	F:CTTCATGGGGAAGGGCGTC
	R:GACACTCCCAGGATGGCATT
P2y6	F:CCTGTCTACTCGCTACCTGC
	R:AAGGCTATGAAGGGCAGCAA
Itga6	F:TGCAGAGGGCGAACAGAAC
	R:GCACACGTCACCACTTTGC
Rac1	F:GAGACGGAGCTGTTGGTAAA
	R:ATAGGCCCAGATTCACTGGTT
Rho	F:AGCTTGTGGTAAGACATGCTTG
	R:GTGTCCCATAAAGCCAACTCTAC
Paxillin	F:GGCATCCCAGAAAATAACACTCC
	R:GCCCTGCATCTTGAAATCTGA
Fak	F:AACGGTCCCCTGGTGCAA
	R:TGACTGAGGCGAAATCCATAGC
Pax7	F:TGGGGTCTTCATCAACGGTC
	R:ATCGGCACAGAATCTTGGAGA
Pcna	F:GGGTGAAGTTTTCTGCAAGTG
	R:GTACCTCAGAGCAAACGTTAGG
Ki67	F:TGCCCGACCCTACAAAATG
	R:GAGCCTGTATCACTCATCTGC
Adamst5	F:TGGCAGCACCAACATAACCA
	R:GCCCACATAAATCCTCTCGG

Plxdc2	F:CCCGGAAGAGGTACAGTCAAA
	R:CCTGAATTGCATGGTGGTCG
Gapdh	F:CCTGTTGCTGTAGCCGTATT
	R:CATCAAGAAGGTGGTGAAGC
U6	F:GCTTCGGCAGCACATATACTAAAAT
	R:CGCTTCACGAATTTGCGTGTCAT
miR-200c-5p	RT:GTCGTATCCAGTGCAGGGTCCGAGGTATTC GCACTGGATACGACCCAAAC
	F:GCGCGTCTTACCCAGCAGT
	R:AGTGCAGGGTCCGAGGTATT

F: forward, R: reverse. U6 and *Gapdh* were used as endogenous control genes for miRNA and mRNA, respectively.
